# Correlation of Plasma Prolactin and Carcinoembryonic Antigen in Colorectal Cancer

**DOI:** 10.7759/cureus.80285

**Published:** 2025-03-09

**Authors:** Parang Aseri, Gayatri Chitara, Mahendra Choudhary

**Affiliations:** 1 General Surgery, Dr. Sampurnanand (SN) Medical College, Jodhpur, IND; 2 Anaesthesiology, Dr. Sampurnanand (SN) Medical College, Jodhpur, IND

**Keywords:** cancer, cea, colon, colorectal, prolactin, rectum

## Abstract

Background

Carcinoembryonic antigen (CEA) has been widely used as a prognostic marker in colorectal cancer for decades. Elevated levels of prolactin have also been observed in various cancers, including CRC; however, the role of elevated prolactin in colorectal cancer remains unclear.

Objective

This study aimed to investigate the correlation between plasma prolactin and CEA levels in patients with colorectal cancer.

Materials and methods

In this observational study, 80 colorectal cancer patients were included. Serum levels of prolactin and CEA were measured using chemiluminescence assay (Siemens AdviaCentaur XP, Siemens Healthineers, Erlangen, Germany). Statistical analysis was performed using the chi-square test, unpaired t-test, and analysis of variance (ANOVA) to evaluate correlations between the two biomarkers.

Results

Our results showed elevated levels of both plasma prolactin and CEA in colorectal cancer patients. A positive correlation was found between serum prolactin and CEA levels in the patient population.

Conclusion

The present study found elevated serum prolactin levels in most patients with raised CEA levels. Further research is required to better understand the role of prolactin as a potential prognostic factor in colorectal cancer and to establish the relationship between serum prolactin and CEA.

## Introduction

Colorectal cancer (CRC) is one of the most common malignancies worldwide. It is the third most common cancer in both men and women, with approximately 663,000 cases, accounting for 10.0% of all cancer cases. In addition, CRC causes about 571,000 deaths, representing 9.4% of all cancer-related deaths [1]. Nearly 60% of CRC cases occur in developed countries. Globally, CRC is estimated to account for approximately 8% of all cancer deaths, contributing to around 608,000 deaths annually. Among cancer-related deaths, CRC is the fourth leading cause of cancer mortality.

In India, the annual incidence rate for colon and rectal cancer in men is 4.4 and 4.1 per lakh, respectively. For women, the annual incidence rate for colon cancer is 3.9 per lakh. The risk of developing invasive colorectal cancer increases with advancing age, with patients aged 50 years and older accounting for more than 90% of new cases. Carcinoembryonic antigen (CEA) has been used as a prognostic marker in CRC for the past three decades [2,3].

Prolactin is a hormone with various biological actions, primarily synthesized by the anterior pituitary gland, and is best known for its role in the mammary glands [4]. Prolactin is also secreted by other normal tissues and human tumors, including malignant tumors of the lung, kidney, uterus, ovary, and breast [5-10]. In addition, prolactin exerts its effects on other cells and tissues such as decidual cells of the placenta, bone, brain, lymphocytes, and breast epithelial cells [11,12].

Previous studies have shown elevated prolactin levels in CRC patients. In a study by Soroush et al., hyperprolactinemia was observed in CRC patients [12]. Similarly, Bhatavdekar et al. also reported higher pre-operative prolactin levels in CRC patients [13]. However, studies by Indinnimeo et al. and Carlson et al. did not find hyperprolactinemia in CRC patients [14,15].

This study aims to determine whether plasma prolactin is elevated in patients with CRC and to compare the levels of plasma CEA and prolactin in these patients.

## Materials and methods

This study was conducted in accordance with the protocol approved by the Medical Ethics Committee of SMS Hospital, Jaipur. After applying inclusion and exclusion criteria, and confirming the cases through clinical examination, radiological investigation, and cytological investigation, the sample population was selected. Written informed consent was obtained from all patients before they participated in the study, ensuring ethical compliance and understanding of the procedures involved.

In this cross-sectional study, 80 colorectal carcinoma (CRC) patients admitted to SMS Hospital, Jaipur, were included. Patients had not been on medications known to increase plasma prolactin levels in the six months prior to the study and did not have any endocrine, renal, or psychiatric disorders that could confound the results. Blood samples were collected between 8:00 am and 10:00 am to minimize diurnal variation, which can affect prolactin levels. Serum prolactin and serum carcinoembryonic antigen (CEA) levels were measured using the Siemens AdviaCentaur XP chemiluminescence assay (Siemens Healthineers, Erlangen, Germany). The cutoff for CEA levels was set at 5 ng/ml based on standard clinical guidelines.

The study involved a thorough clinical evaluation of all patients, including a detailed history and physical examination. Blood samples for prolactin and CEA measurement were collected after the initial assessment. The blood draws were timed to avoid any influence from circadian fluctuations in hormone levels, ensuring the reliability of the data.

Descriptive statistics were used to present qualitative data as percentages and proportions while quantitative data were expressed as mean ± standard deviation (SD). Differences in proportions were assessed using the chi-square test, and differences in means were evaluated with the unpaired t-test or analysis of variance (ANOVA) as appropriate. A p-value of <0.05 was considered statistically significant, indicating that the differences observed in the data were unlikely to be due to random chance.

In conclusion, this study aimed to evaluate the relationship between serum prolactin and CEA levels in colorectal carcinoma patients, with a focus on minimizing external factors that could skew the results. The use of stringent inclusion/exclusion criteria, proper timing of blood collection, and appropriate statistical methods ensured that the findings were both valid and reliable.

## Results

**Figure 1:**
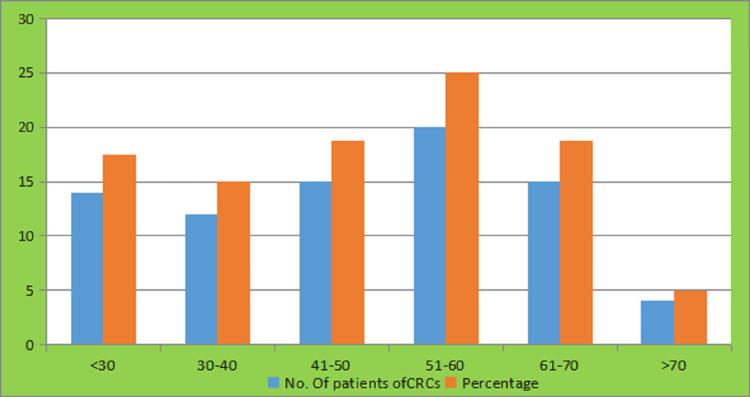
Age distribution of patients

In our study, 80 colorectal carcinoma (CRC) patients were included, of which 52 were male and 28 were female. The average age of presentation was 48.94 ± 15.11 years. Among the 80 cases, 43 (53.75%) were diagnosed with carcinoma of the rectum while 37 (46.25%) had colon cancer (Figure 1).

**Figure 2:**
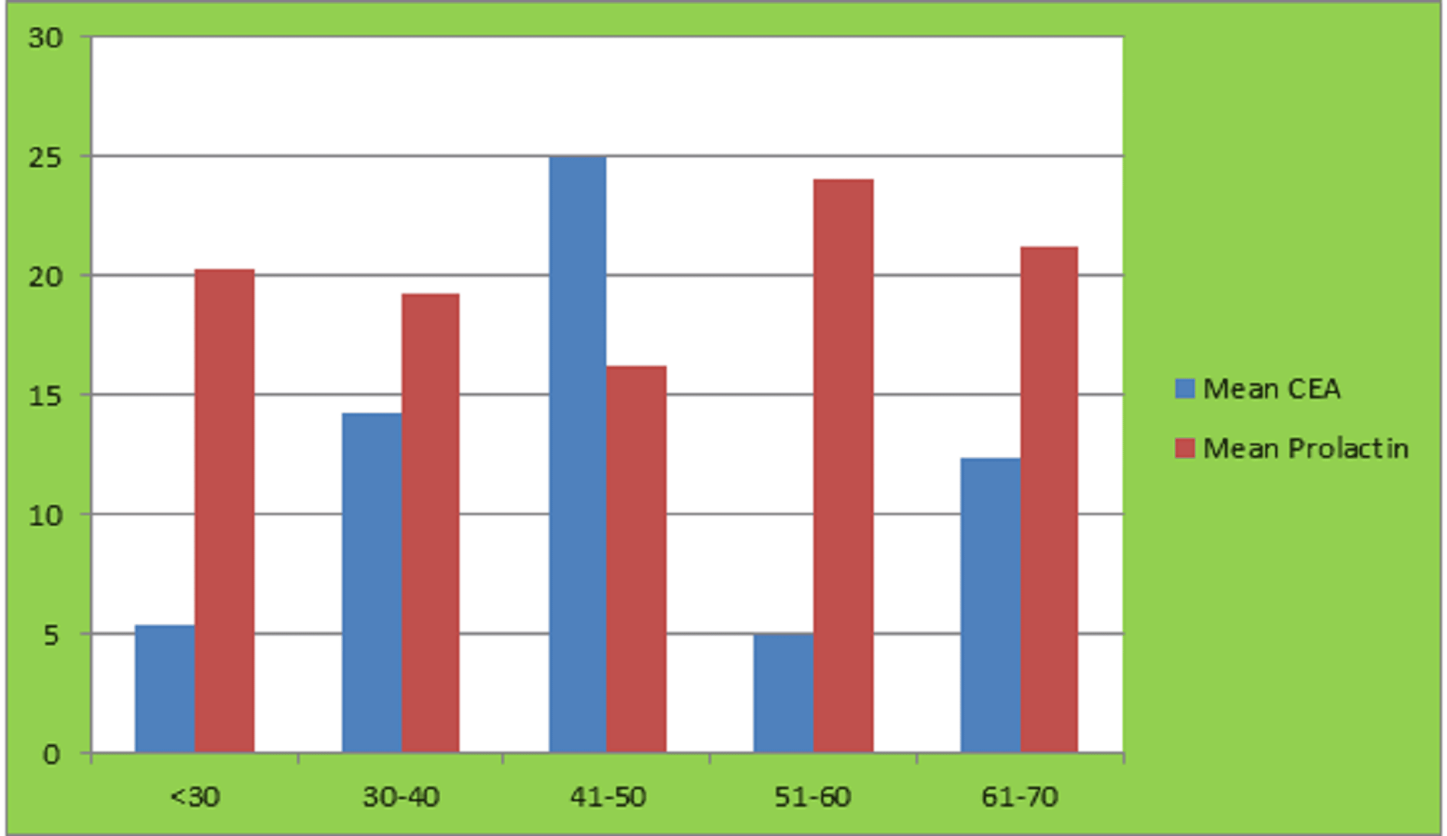
Distribution of CEA and prolactin in patients CEA: carcinoembryonic antigen

The mean serum CEA and prolactin levels were compared between male and female patients. In males, the mean CEA level was 14.31 while the mean prolactin level was 20.8. In females, the mean CEA level was 6.6, and the mean prolactin level was 18.47. These findings suggest that both CEA and prolactin levels were higher in males as compared to females (Figure 2).

To compare serum CEA and serum prolactin in all age groups, the ANOVA test was carried out to test the null hypothesis stating that there is no difference in mean serum CEA and mean serum prolactin in all age groups. The findings from Table 1 state that the mean serum CEA and serum prolactin were statistically insignificant across the age groups, as the p-value was >0.05.

**Table 1:** Comparison of CEA and prolactin according to age group CEA: carcinoembryonic antigen

Age Group	Mean CEA	Mean Prolactin
<30	5.38±6.1	20.24±26.41
30-40	14.21±26.97	19.25±25.19
41-50	24.94±33.78	16.19±14.07
51-60	5.01±6.38	24.08±17.43
61-70	12.39±19.38	21.22±21.21
>70	1.7±0.98	10.7±13.54
P-value	0.082	0.819

Regarding serum CEA and prolactin levels, significant elevations were observed in patients with CRC. Hyperprolactinemia was found in 34 (42.5%) patients while elevated CEA levels were seen in 45 (56.25%) patients. The mean CEA level in CRC patients in our study was 11.61. Notably, patients with carcinoma of the rectum had a higher mean CEA level (18.47) compared to those with colon cancer (10.62). A CEA value greater than 3 ng/ml was found in 45 patients (56.25%), which is above the normal threshold of 3 ng/ml. In contrast, the mean prolactin level in CRC patients was 19.98, with higher levels observed in colon cancer (13.26) compared to rectal cancer (10.19). Elevated prolactin (>18 ng/ml) was observed in 34 patients (42.5%).

A comparison between CEA and prolactin levels revealed that when CEA levels were below 3 ng/ml, the mean prolactin level was lower (12.05). However, when CEA levels were above 3 ng/ml, the mean prolactin level increased significantly to 22.65. This indicates a positive association between elevated CEA and prolactin levels in CRC patients. Additionally, the mean CEA level was highest in the 41-50 year age group while elevated prolactin levels were more common in the 51-60 year age group, with T-value = 9.16, p-value = 0.003(s).

The T-test was carried out to test the null hypothesis stating that there is a positive correlation of serum prolactin in patients of CRCs. The findings from Table 2 state that the mean serum prolactin level is statistically significant in patients with colorectal cancer as p-value = 0.003. The mean serum prolactin level is 19.98±20.14 in patients with CRC. When compared to serum CEA, its mean value is 11.61±21.35.

**Table 2:** Mean CEA vs mean prolactin CEA: carcinoembryonic antigen

CEA level	Prolactin level	
	Mean	SD
<3	12.05	10.19
>3	22.65	14.93

**Figure 3:**
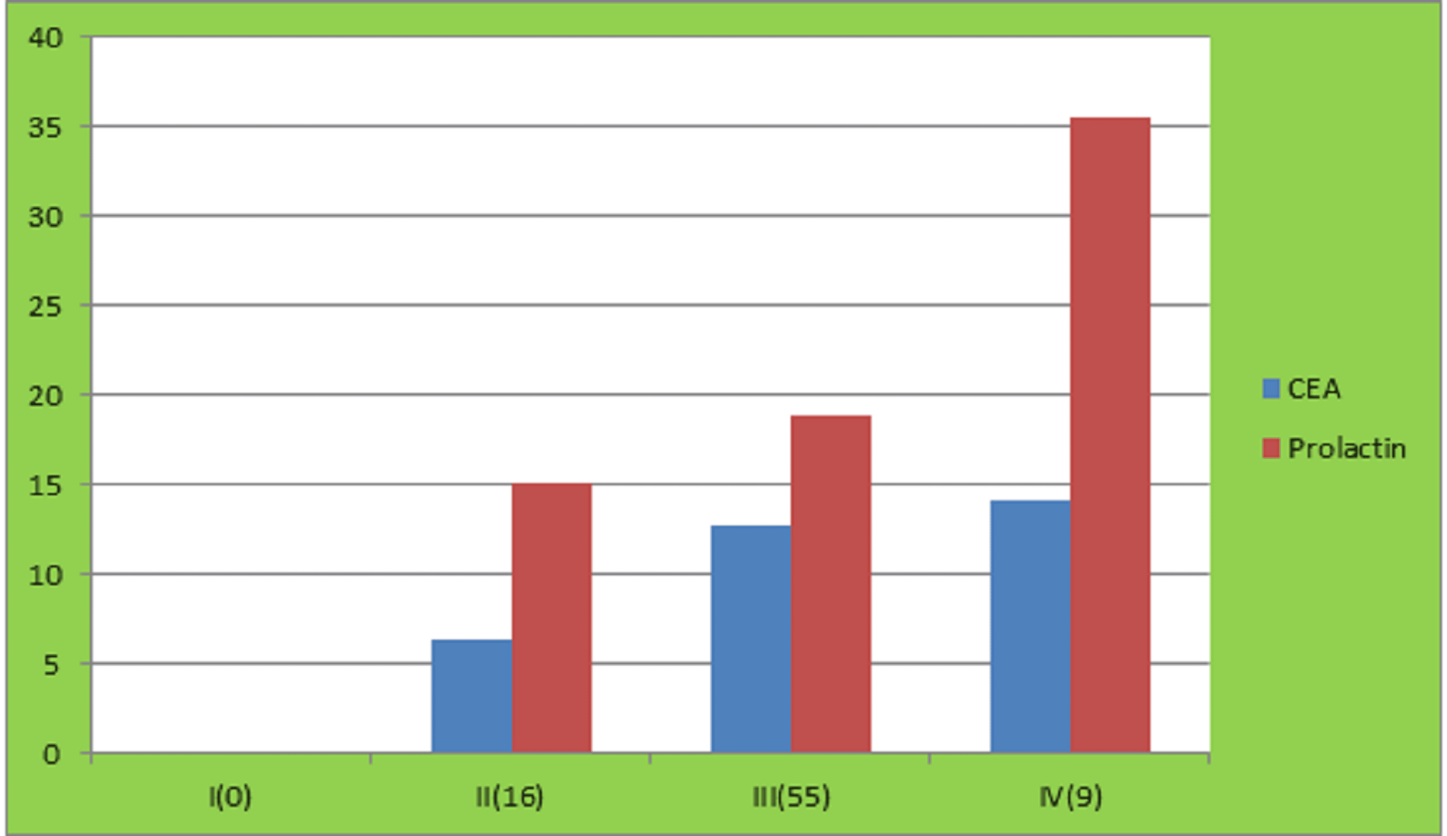
Distribution of CEA and prolactin according to stage CEA: carcinoembryonic antigen

In terms of disease stage, the majority of patients presented at stage III (n=55, 68.75%). Stage II accounted for 16 patients (20%), and stage IV for 9 patients (11.25%). The mean CEA and prolactin levels were highest in stage IV patients (CEA = 14.18, prolactin = 35.49), followed by stage III (CEA = 12.72, prolactin = 18.85) and stage II (CEA = 6.34, prolactin = 15.16). These results indicate that both CEA and prolactin levels increase with advancing disease stage (Figure 3).

**Figure 4:**
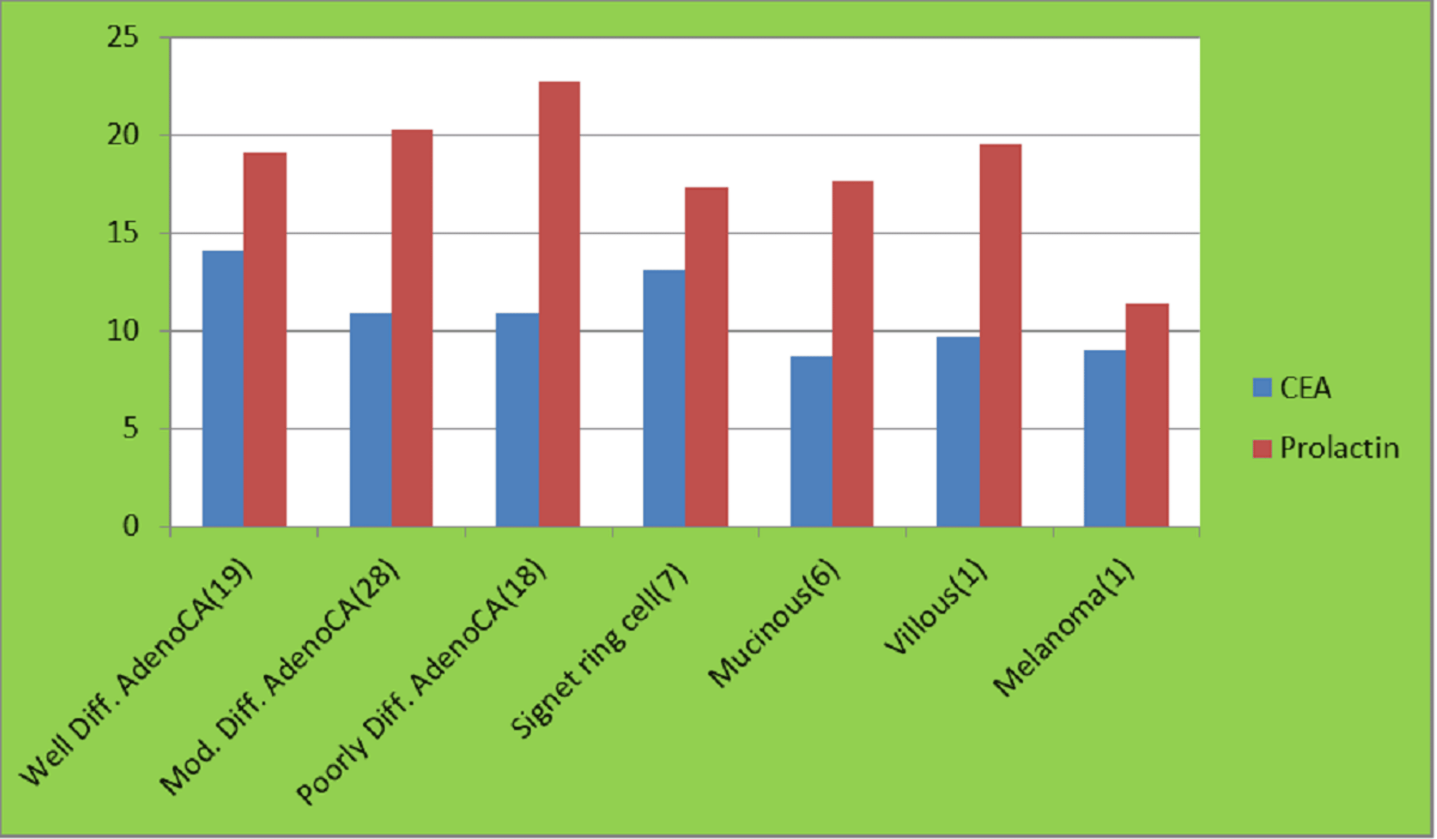
Distribution of CEA and prolactin according to histological type CEA: carcinoembryonic antigen

Histopathological examination revealed that the majority of patients had moderately differentiated adenocarcinoma (n=28, 35%), followed by well-differentiated adenocarcinoma (n=19, 23.75%), poorly differentiated adenocarcinoma (n=18, 22.5%), mucinous adenocarcinoma (n=6, 7.5%), and a few cases of villous type and melanoma (1.25% each) (Figure 4).

Regarding lifestyle factors, 47.5% of patients were smokers while 52.5% were non-smokers. This finding suggests that factors other than smoking also contribute to the development of colorectal cancer, as a significant proportion of CRC patients were non-smokers.

## Discussion

Prognostic factors play an important role in the management of carcinoma. For the assessment of therapeutic response and follow-up, tumor markers are valuable tools. Many tumors, even in the sub-clinical stage, can be diagnosed by evaluating tumor markers. Ectopic secretion of hormones, such as prolactin, by non-endocrine neoplasms is recognized and is often used for therapeutic monitoring. Prolactin is primarily secreted by the pituitary gland, but recent studies have shown elevated prolactin levels in malignancies of the breast, lung, prostate, and ovary [16].

Elevated prolactin levels have also been observed in patients with colorectal cancer (CRC). Soroush et al. found a positive correlation between prolactin levels and colorectal cancer, suggesting its potential role in this disease [12]. Bhatavdekar et al. also reported elevated pre-operative prolactin levels in patients with colorectal cancer [13]. Ilan et al. observed raised prolactin levels in 53% of patients with colorectal malignancies [17]. However, some studies have not supported this finding. Indinnimeo et al. did not observe positive prolactin immunostaining or hyperprolactinemia in patients with colorectal cancer [14]. Similarly, Baert et al. in a series of 32 patients found no pre-operative hyperprolactinemia and their study did not support the hypothesis of ectopic prolactin production in colorectal cancer [18].

Previous studies that reported normal levels of prolactin suggested that factors such as renal, endocrine, and psychiatric disorders, medications, and premenopausal status could contribute to hyperprolactinemia in patients with colorectal neoplasms [15,18]. In our study, such factors were excluded, and our results indicated a positive correlation between serum prolactin and the presence of colorectal cancer. This finding suggests that prolactin could be a useful biomarker for colorectal cancer, especially in the absence of confounding factors.

CEA is a set of highly related glycoproteins involved in cell adhesion. CEA production normally occurs in gastrointestinal tissue during fetal development but ceases before birth. In healthy adults, very low levels of CEA are present. However, in some types of cancer, the serum level of CEA is elevated, making it a valuable tumor marker in clinical tests. Over-expression of CEA is commonly observed in colorectal cancer and is used as a tumor marker for the disease. Patients with advanced disease, either locally or with distant metastasis, tend to have higher CEA levels preoperatively. Preoperative estimation of serum CEA levels can serve as a useful predictive factor for the outcome of surgical operations [2,3,12,16].

In our study, we found elevated levels of both plasma prolactin and CEA in patients with colorectal cancer. Our results suggest that prolactin may also serve as a valuable tumor marker for CRC. Given that the laboratory costs for detecting prolactin are generally lower than those for CEA, prolactin could be a cost-effective alternative in CRC diagnosis and monitoring. However, further studies are needed to validate the role of prolactin as a tumor marker for colorectal cancer and to explore its potential clinical applications.

## Conclusions

Carcinoembryonic antigen (CEA) is an important tumor marker, as it is expressed in most cases of colorectal cancer (CRC). Higher CEA levels generally indicate advanced disease, making it a crucial tool for monitoring CRC and identifying recurrence. In the present study, we observed an increase in serum prolactin levels in almost all patients where CEA levels were elevated. Considering that the laboratory cost for detecting CEA is higher than that for prolactin, prolactin could serve as a valuable alternative marker for CRC.

However, further research is required to better understand prolactin's role as a prognostic factor and to explore the potential correlation between serum prolactin and CEA. Additionally, prolactin receptor antagonists and neutralizing antibodies could offer a new form of therapy for resistant colorectal cancer. On the genetic level, constructing a prolactin receptor single nucleotide polymorphism (SNP) risk profile for affected patients could enable more personalized treatment strategies in the future.

## References

[1] (2008). GLOBOCAN. Colorectal cancer: estimated incidence, mortality, and prevalence worldwide in 2008. http://globocan.iarc.fr/factsheets/cancers/colorectal.asp.

[2] Adams WJ, Morris DL (1996). Carcinoembryonic antigen in the evaluation of therapy of primary and metastatic colorectal cancer. Aust N Z J Surg.

[3] Konishi F (2002). CEA doubling time and CEA half-life in the prediction of recurrences after colorectal cancer surgery. Jpn J Clin Oncol.

[4] Ben-Jonathan N, Mershon JL, Allen DL, Steinmetz RW (1996). Extrapituitary prolactin: distribution, regulation, functions, and clinical aspects. Endocr Rev.

[5] Rees LH, Bloomfield GA, Rees GM, Corrin B, Franks LM, Ratcliffe JG (1974). Multiple hormones in a bronchial tumor. J Clin Endocrinol Metab.

[6] Stanisic TH, Donovan J (1986). Prolactin secreting renal cell carcinoma. J Urol.

[7] Hsu CT, Yu MH, Lee CY, Jong HL, Yeh MY (1992). Ectopic production of prolactin in uterine cervical carcinoma. Gynecol Oncol.

[8] Hoffman WH, Gala RR, Kovacs K, Subramanian MG (1987). Ectopic prolactin secretion from a gonadoblastoma. Cancer.

[9] Mujagić Z, Mujagić H (2004). Importance of serum prolactin determination in metastatic breast cancer patients. Croat Med J.

[10] Dugan AL, Schwemberger S, Babcock GF, Buckley D, Buckley AR, Ogle CK, Horseman ND (2004). Effects of prolactin level on burn-induced aberrations in myelopoiesis. Shock.

[11] Llovera M, Touraine P, Kelly PA, Goffin V (2000). Involvement of prolactin in breast cancer: redefining the molecular targets. Exp Gerontol.

[12] Soroush AR, Zadeh HM, Moemeni M, Shakiba B, Elmi S (2004). Plasma prolactin in patients with colorectal cancer. BMC Cancer.

[13] Bhatavdekar JM, Patel DD, Chikhlikar PR, Shah NG, Vora HH, Ghosh N, Trivedi TI (2001). Ectopic production of prolactin by colorectal adenocarcinoma. Dis Colon Rectum.

[14] Indinnimeo M, Cicchini C, Memeo L (2001). Plasma and tissue prolactin detection in colon carcinoma. Oncol Rep.

[15] Carlson HE, Zarrabi MH, Lyubsky SL (2000). Lack of association between hyperprolactinemia and colon carcinoma. Cancer Invest.

[16] Maestranzi S, Przemioslo R, Mitchell H, Sherwood RA (1998). The effect of benign and malignant liver disease on the tumour markers CA19-9 and CEA. Ann Clin Biochem.

[17] Ilan Y, Sibirsky O, Livni N, Gofrit O, Barack V, Goldin E (1995). Plasma and tumor prolactin in colorectal cancer patients. Dig Dis Sci.

[18] Baert D, Matthys C, Gillardin JP (1998). Prolactin and colorectal cancer: is there a connection?. Acta Gastroenterol Belg.

